# Local chemical heterogeneity enabled superior zero thermal expansion in nonstoichiometric pyrochlore magnets

**DOI:** 10.1093/nsr/nwae462

**Published:** 2024-12-17

**Authors:** Yanming Sun, Ruohan Yu, Sergii Khmelevskyi, Kenichi Kato, Yili Cao, Shixin Hu, Maxim Avdeev, Chin-Wei Wang, Chengyi Yu, Qiang Li, Kun Lin, Xiaojun Kuang, Xianran Xing

**Affiliations:** Beijin g Advanced Innovation Center for Materials Genome Engineering, Institute of Solid State Chemistry, Department of Physical Chemistry, University of Science and Technology Beijing, Beijing 100083; The Sanya Science and Education Innovation Park of Wuhan University of Technology, Sanya 572000; Vienna Scientific Cluster Research Center, Technical University of Vienna, Vienna A-1040; RIKEN SPring-8 Center, Hyogo; Beijin g Advanced Innovation Center for Materials Genome Engineering, Institute of Solid State Chemistry, Department of Physical Chemistry, University of Science and Technology Beijing, Beijing 100083; Institute of Applied Magnetics, Key Laboratory for Magnetism and Magnetic Materials of the Ministry of Education, Lanzhou University, Lanzhou 730000; Australian Nuclear Science and Technology Organisation, Lucas Heights, New South Wales 2234; School of Chemistry, The University of Sydney, Sydney, New South Wales 2006; Neutron Group, Synchrotron Radiation Research Center, Hsinchu 30076; Beijin g Advanced Innovation Center for Materials Genome Engineering, Institute of Solid State Chemistry, Department of Physical Chemistry, University of Science and Technology Beijing, Beijing 100083; Beijin g Advanced Innovation Center for Materials Genome Engineering, Institute of Solid State Chemistry, Department of Physical Chemistry, University of Science and Technology Beijing, Beijing 100083; Beijin g Advanced Innovation Center for Materials Genome Engineering, Institute of Solid State Chemistry, Department of Physical Chemistry, University of Science and Technology Beijing, Beijing 100083; Guangxi Key Laboratory of Electrochemical and magnetochemical Functional Materials, College of Chemistry and Bioengineering, Guilin University of Technology, Guilin 541004; Beijin g Advanced Innovation Center for Materials Genome Engineering, Institute of Solid State Chemistry, Department of Physical Chemistry, University of Science and Technology Beijing, Beijing 100083

**Keywords:** zero thermal expansion, pyrochlore, magnetic structure, local composition heterogeneity

## Abstract

The design of zero thermal expansion (ZTE) materials is urgently required as dimension-stable components in widespread modern high-precision technologies. Local chemical order has been of great importance in engineering advanced inorganic materials, but its role in optimizing the ZTE is often overlooked. Herein, we propose local composition heterogeneity for developing superior ZTE via a nonstoichiometric strategy. A remarkably low coefficient of thermal expansion of *α_a_* = +1.07 × 10^−6^ K^−1^ is achieved from 3 to 440 K in a quaternary Zr-Nb-Fe-Co pyrochlore magnet, which is the widest temperature range among known cubic ZTE metals. High-resolution synchrotron X-ray diffraction and magnetization measurements reveal that all the Bragg peaks split as resulting from two cubic phases with different magnetic orders. Scanning transmission electron microscopy, Mössbauer spectroscopy and theoretical calculations indicate that such phase separation intimately derives from excess Co dopant preferentially clustering on the Fe pyrochlore-lattice (16*d*) and simultaneously yielding an antisite Fe on Zr/Nb sublattice (8*a*). The Co content in pyrochlore-lattice has weaker exchange interactions than that of Fe, but the antisite Fe introduces extra positive exchange interactions between 8*a* and 16*d* sites. Local composition fluctuation of Co and Fe thus affects the interplanar ferromagnetic order of pyrochlore-lattice and balances the normal phonon effect successively on heating. Superior corrosion resistance to both acid and alkaline conditions merits potential applications of the present ZTE metal.

## Teaser

This work proposes local composition heterogeneity for developing superior zero thermal expansion (ZTE) in pyrochlore magnet via a nonstoichiometric strategy, and the role of local chemical ordering in optimizing the engineering of materials for ZTE is further recognized.

## INTRODUCTION

Zero thermal expansion (ZTE) materials, because they are size-stable with temperature, are among the fundamental components in advanced modern technologies of the aerospace and semiconductor industry [1–3]. To date, the number of ZTE materials is rare in nature, and most of these are elaborately designed by taking advantage of negative thermal expansion materials that have been found in a variety of oxides [4], fluorides [5], alloys [6] and metal-organic frameworks (MOFs) [7], etc. Chemical manipulations, including element substitutions, size effect and ion intercalation, have been employed to regulate diverse order parameters such as phonon vibration, polarization, magnetic and charge order [8–10]. Among them, the ZTE metals are capable of magnetism [11,12], thermal [13–15] and electric conductivity [16,17], as well as possessing mechanical properties [18], thereby proving to be of the highest merit [19,20]. For practical use, cubic crystals are essential, especially for ZTE metals, because the synthesis of bulk metallic materials typically accompanies the crystalline texture that is detrimental to the ZTE of anisotropic crystal in macroscale [18].

At the atomic level, compositional heterogeneity seems to be a prerequisite for changing lattice order parameters, thus tuning thermal expansion. However, local composition fluctuations, such as local chemical order, short-range symmetry and elemental cluster, may have their own order parameters and play a significant role in the ZTE, but are frequently simplified by dealing with a disordered model thus far, in which heterogeneous atoms separate randomly in space. For example, the role of increasing Ge content in tetragonal distortions of cubic Mn_3_Cu_0.5_Ge_0.5_N with giant negative thermal expansion remains unclear [21]; chemical order is crucial for stable cubic structure in double ReO_3_-fluorides, but local chemical order in doped-ScF_3_ ZTE materials is always overlooked [5]; diverse local polar structures were observed in multicomponent polar oxides and significantly affect ferroelectric properties [22], but the impact on ZTE has been scarcely studied. A thorough comprehension of the correlation between local composition heterogeneity and thermal expansion, whether they interact with each other, and by which thermal expansion can be well manipulated, is not only important for fundamental knowledge on the coupling of multiple degrees of freedom, but would also benefit in designing practical ZTE materials.

**Figure 1.**
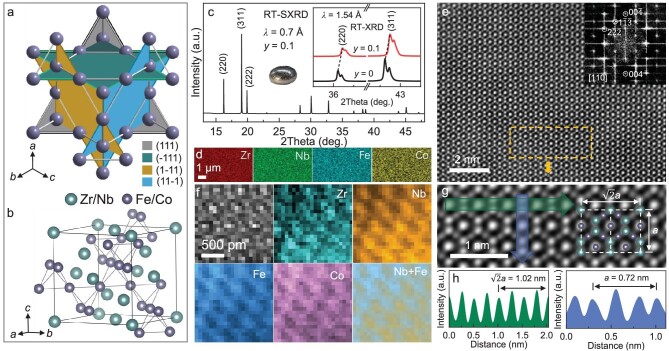
(a) The structure of (Zr, Nb)Fe_2_Co*_y_* with the four dihedral kagome planes; (b) alternative view of the cubic crystal structure of (Zr, Nb)Fe_2_Co*_y_* in a pyrochlore lattice; (c) SXRD result of *y* = 0.1 at 300 K (the inset shows XRD results for *y* = 0 and 0.1 in the laboratory, together with a picture of the ingot); (d) EDS elemental mapping of the *y* = 0.1; (e) HAADF-STEM image along the [110] zone axis of *y* = 0.1 (the inset shows the corresponding fast Fourier transform (FFT) profile); (f) the STEM images and EELS mapping along the [110] zone axis of *y* = 0.1; (g) the enlarged view of the yellow dashed area in (e); the inset shows the cubic structure model; (h) intensity profiles along the green and blue arrow directions marked in (g), respectively.

Pyrochlore lattice is a 3D cubic network made up of corner-sharing tetrahedra (Fig. 1a) [23]. Strong coupling between cubic lattice geometry and spin provides fertile ground for exploring anomalous thermal expansion with a magnetic order [8,24]. A representative example is C15 Laves phase intermetallic compound (space group: *Fd*-3*m*) including *R*Co_2_ (*R* = rare earth) and ZrFe_2_ series (Fig. 1b) [25–31]. Many attempts of elemental substitutions have been employed to tailor the transition from first to second order and engender the ZTE [27,28,32]. However, to the former, negative thermal expansion was found near the magnetic phase transition temperature. These ZTEs were generally below room temperature and a narrow operating window has been a major concern [2,18,33]. In regard to the latter, it was found that although high Nb/Ta substitution for Zr introduces chemical pressure on the pyrochlore lattice of Fe, and tunes the magnetic order and thermal expansion [26,29], so far eliminating hexagonal impurity excessively still relies on a high-temperature synthesis condition [34]. Recently, a nonstoichiometric strategy has emerged as another route to introduce extra magnetic exchange interaction and regulate magnetic order and thus the ZTE over a wide temperature window, such as HoCo_2_Mn_0.5_ and (Zr, Nb)Fe_2+_*_x_* [34,35]. Nonstoichiometry of other excess 3*d* transition metals such as Co and Ni on controlling thermal expansion has not been reported to date.

In the present work, we report on an isotropic ZTE of *α_a_* = +1.07 × 10^−6^ K^−1^ from 3 to 440 K in a nonstoichiometric pyrochlore magnet Zr_0.75_Nb_0.25_Fe_2_Co_0.1_ with excess Co doping. A careful atomic crystal structure evidenced by scanning transmission electron microscopy shows the preferred clustering of Co atoms on pyrochlore-lattice substituted for Fe and ultimately leading to an antisite Fe on Zr/Nb-sublattice. *In-situ* high-resolution synchrotron X-ray diffraction, neutron powder diffraction, Mössbauer spectroscopy and *ab initio* calculations reveal that local composition heterogeneity derived from Co dopant regulated the magnetic order and thus contribute to such ultrawide ZTE. Superior corrosion resistance to both acid and alkaline conditions indicate that such ZTE metals merit applications in the future.

## RESULTS AND DISCUSSION

### Crystal structure

All the present Zr_1−_*_x_*Nb*_x_*Fe_2_Co*_y_* samples were prepared using the arc-melting method and silver ingot indicates good metallicity (see the inset of Fig. 1c). To better observe the role of Co in magnetic order and thermal expansion, we explored three sets of samples: Zr_0.85_Nb_0.15_Fe_2_Co*_y_* (*y* = 0, 0.05, 0.1, 0.15 and 0.2), Zr_0.75_Nb_0.25_Fe_2_Co*_y_* (*y* = 0, 0.1, 0.2 and 0.3), and Zr_0.65_Nb_0.35_Fe_2_Co*_y_* (*y* = 0, 0.05, 0.1, 0.15 and 0.2), respectively (see Figs S1–S3 in the Supplementary data for details). The as-prepared Zr_0.75_Nb_0.25_Fe_2_Co_0.1_ was adopted for a detailed structure study. As shown in Fig. 1c, room-temperature synchrotron X-ray powder diffraction (SXRD) indicates cubic symmetry. X-ray energy dispersive spectroscopy (EDS) elemental analysis shows that the four elements were determined to be homogeneously distributed at a macroscopic level (Fig. 1d and Fig. S4). The high-angle annular dark-field (HAADF) pattern also confirms a perfect cubic symmetry of Zr_0.75_Nb_0.25_Fe_2_Co_0.1_ with negligible defects in the local structure (Fig. 1e). As shown in Fig. 1f, electron energy loss spectroscopy (EELS) further evidences that the Co content was introduced into the (Zr, Nb)Fe_2_ matrix and clearly occupies the pyrochlore lattice. The intensity variation may indicate that there might be local compositional inhomogeneity (discussed later). The enlarged atomic image shows that the lattice parameter *a* of such cubic phase is ∼7.2 Å, which is similar to that of SXRD 7.00(1) Å at 300 K.

### Thermal expansion

**Figure 2.**
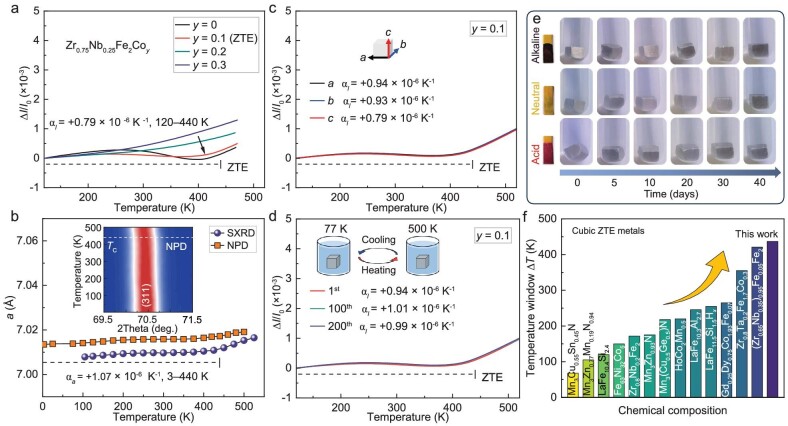
(a) Linear thermal expansion (Δ*I*/*I*_0_) for Zr_0.75_Nb_0.25_Fe_2_Co*_y_* (*y* = 0, 0.1, 0.2 and 0.3). (b) Temperature dependence of SXRD and NPD-measured lattice parameters of *y* = 0.1 (the inset shows a contour NPD plot of the peak (311) in *y* = 0.1). (c) Linear thermal expansions of *y* = 0.1 along three directions. (d) The dilatometer thermal expansions of *y* = 0.1 in the first, 100th and 200th cycles (the inset shows the cyclic thermal shock experiment in the 77–500 K temperature window). (e) Photographs of *y* = 0.1 immersed in 3.36 mol/L hydrochloric acid solution (acid, bottom), 0.6 mol/L sodium chloride solution (neutral, middle) and 1 mol/L of sodium hydroxide solution (alkaline, top) for different days. (f) Temperature windows for typical isotropic cubic ZTE metals.

Apparent linear thermal expansion was measured for Zr_0.75_Nb_0.25_Fe_2_Co*_y_* (*y* = 0, 0.1, 0.2 and 0.3), as shown in Fig. 2a. Zr_0.75_Nb_0.25_Fe_2_ initially shows a low positive thermal expansion (PTE, *ᾱ_l_* = +1.75 × 10^−6^ K^−1^, 120–279 K) that then turns to negative thermal expansion (NTE; *ᾱ_l_* = −2.41 × 10^−6^ K^−1^, 279–407 K). With Co doping, the NTE gradually vanishes. Intriguingly, a ZTE behavior is determined in Zr_0.75_Nb_0.25_Fe_2_Co_0.1_ from 120–440 K, *ᾱ_l_* = +0.79 × 10^−6^ K^−1^. A remarkably low coefficient of thermal expansion is found from 213–400 K across room temperature (*ᾱ_l_* = −0.08 × 10^−6^ K^−1^). A similar tendency is determined in another two sets of Zr_0.85_Nb_0.15_Fe_2_Co*_y_* and Zr_0.65_Nb_0.35_Fe_2_Co*_y_* (Fig. S5). On the other hand, the lattice parameter *a* determined by neutron powder diffraction and SXRD is plotted as a function of temperature in Fig. 2b. The lattice parameter *a* shows as nearly constant over the investigated magnetic ordering temperature window from 3 to 440 K and demonstrates a ZTE of *ᾱ_a_* = +1.07 × 10^−6^ K^−1^. For a practical step, the isotropic ZTE of the ingot is confirmed in three directions from 120 to 440 K (Fig. 2c): *a* direction: *ᾱ_l_* = +0.94 × 10^−6^ K^−1^; *b* direction: *ᾱ_l_* = +0.93 × 10^−6^ K^−1^; and *c* direction: *ᾱ_l_* = +0.79 × 10^−6^ K^−1^. In addition to thermal cycling stability (Fig. 2d), the present ZTE metals also show excellent corrosion resistance to acid, neutral and alkaline conditions (Fig. 2e). To date, most of the ZTE in cubic metals shows a narrow operating working temperature (Fig. 2f and Table S3) [10,20,21,29,33–41], e.g. Mn_3_Cu_0.5_Ge_0.5_ N (*ᾱ_l_* = +0.1 × 10^−6^ K^−1^, 12–230 K) [21], LaFe_10.4_Si_2.4_ (*ᾱ_l_* = +0.8 × 10^−6^ K^−1^, 15–135 K) [20], and Zr_0.8_Ta_0.2_Fe_1.7_Co_0.3_ (*ᾱ_l_* = +0.21 × 10^−6^ K^−1^, 5–360 K) [33]. The present facile ZTE also covers an ultrawide temperature window up to 440 K, which may benefit its application in the future.

### Magnetization measurements

**Figure 3.**
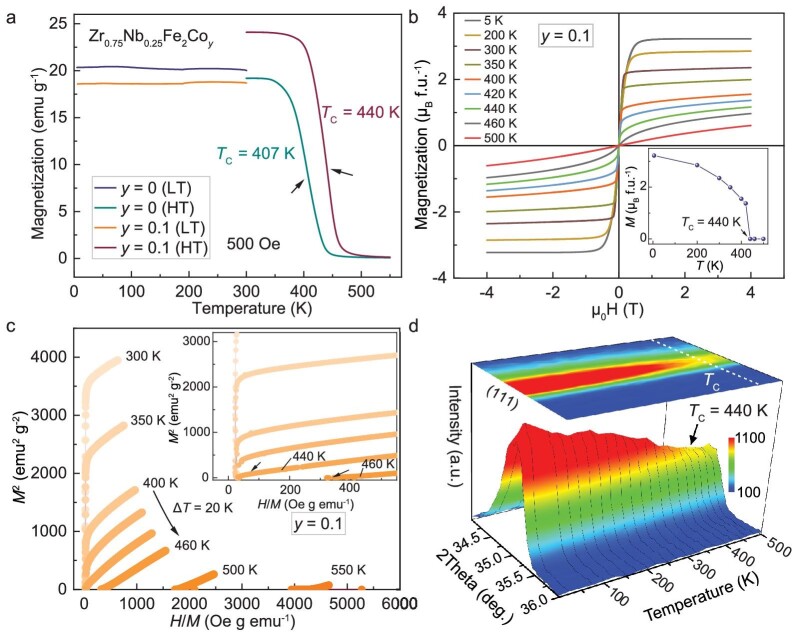
(a) Temperature dependence of magnetization (*M*-*T*) for Zr_0.75_Nb_0.25_Fe_2_Co*_y_* (*y* = 0 and 0.1) in zero-field cooling (ZFC) under an applied magnetic field of 500 Oe. (b) Magnetization as a function of applied magnetic field (*M*-*H*) curves for *y* = 0.1. (c) Isothermal Arrot plot of *y* = 0.1 based on *M*-*H* curves; (d) 3D contour plot of NPD patterns near peak (111) for *y* = 0.1.

The macroscopic magnetic behavior of Zr_0.75_Nb_0.25_Fe_2_ and Zr_0.75_Nb_0.25_Fe_2_Co_0.1_ was studied by the temperature dependence of magnetization (*M*-*T*) in zero-field cooling (ZFC) at a magnetic field of 500 Oe (Fig. 3a and Fig. S6). It was found that there is a distinct ferromagnetic-paramagnetic phase transition in the as-prepared two samples, and that the Curie temperature (*T*_C_) increased from 407 to 440 K. The magnetization as a function of magnetic field (*M*-*H*) curves was measured from 5 to 500 K for Zr_0.75_Nb_0.25_Fe_2_Co_0.1_, as shown in Fig. 3b and Figs S7–S11. An obvious saturation magnetization and low coercive field in all the *M*-*H* curves illustrate the soft ferromagnetic nature of Zr_0.75_Nb_0.25_Fe_2_Co_0.1_ during the magnetic order-disorder transition. Isotherm Arrot plots (*H*/*M* versus *M*^2^) were employed to evaluate the magnetic phase transition (Fig. 3c and Fig. S12) [42]. All the positive slopes clearly evidence second-order phase transition and the *T*_C_ was deduced near 440 K by determining the intercept in the axis of *M*^2^ (see the arrows in Fig. 3c) [43]. Temperature dependence of neutron powder diffraction (NPD) patterns further evidences that the spontaneous magnetic contributions on the Bragg peak (111) disappear upon heating and disappear near 440 K (Fig. 3d). The same transition temperature between the ZTE and magnetic ordering indicates a strong coupling between magnetism and lattice [8].

### Temperature dependence of crystal structure

**Figure 4.**
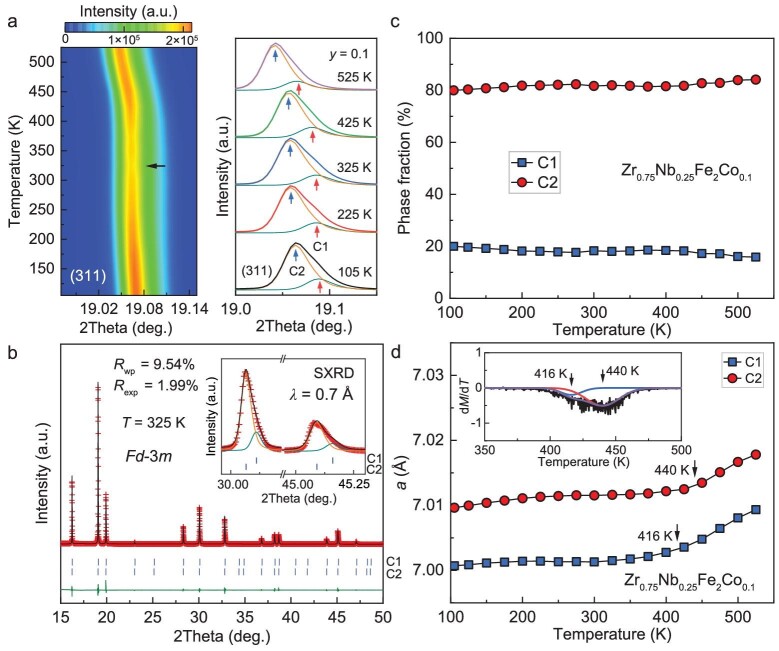
(a) Temperature dependence of synchrotron X-ray diffraction of Zr_0.75_Nb_0.25_Fe_2_Co_0.1_ (*y* = 0.1). (b) Rietveld refinement using two different cubic models of SXRD patterns at 325 K for *y* = 0.1. (c) Phase fraction, and (d) lattice parameters of C1 and C2 phases as a function of temperature determined by SXRD patterns (the inset shows the differential curves of *M*-*T* at 500 Oe).

To determine the evolution of lattice below *T*_C_ more clearly, the temperature dependence of high-resolution SXRD (*λ* = 0.7 Å) was measured from 105 to 525 K, as shown in Fig. 4a. Interestingly, the contour plots show peak broadening near room temperature and a distinct peak tail emerges at 400 K. All the Bragg peaks show similar evolution at the investigated temperatures. Using only one cubic structure cannot fit the patterns well, as shown in Fig. S13. Rietveld refinement using two cubic phases with different lattice parameters can fit the SXRD patterns better (Fig. 4b). In this case, Fig. 4c plots the phase fraction as a function of temperature for these two cubic phases. It can be observed that the fraction changes at all the temperatures: one is near 20% (C1) and the other is 80% (C2), which indicates the phase separation is intrinsic and may be related to compositional heterogeneity. Notably, C1 has a smaller volume and C2 has a larger volume (Fig. 4d). Asymmetric differential curves of *M*-*T* also indicate that there are two magnetic phase transitions and we tried to determine the transition temperatures by using two peaks with the same ratio with SXRD results (inset in Fig. 4d and see Table S4 for details). At the same time, the thermal expansion of the two phases is also different: the thermal expansion anomaly of C1 disappears at lower temperature (∼416 K) and C2’s magnetic ordering temperature (∼440 K) is similar to that determined by *M*-*T* curves (Fig. 3a and inset in Fig. 4d). It has been reported that the Co substitution for Fe in ZrFe_2_-based intermetallic compounds significantly decreases the lattice volume [6]. Thus, a smaller volume of the C1 phase indicates that there may be more Co content than that of the C2 phase. On the other hand, Co content also decreases the *T*_C_ and the thermal expansion of the C1 phase disappears at a lower temperature.

### Hyperfine magnetic structure

**Figure 5.**
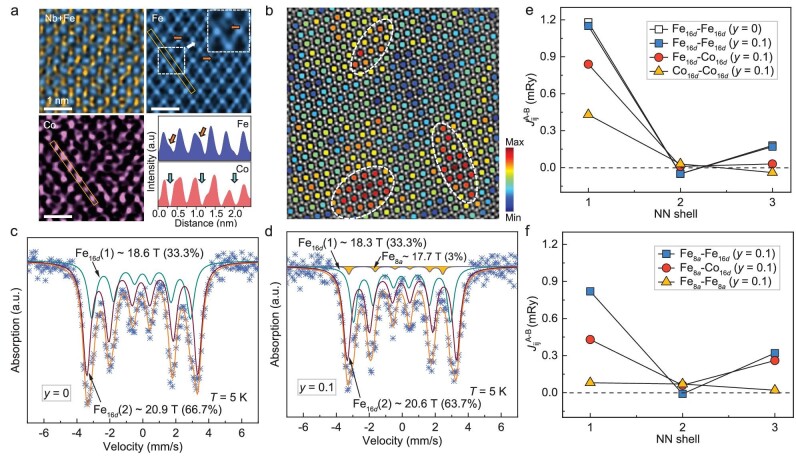
(a) Atomic-resolution energy dispersive X-ray mapping and intensity profile of Zr_0.75_Nb_0.25_Fe_2_Co*_y_* (*y* = 0.1). (b) The integrated intensity profile of the column of Fe atoms in the middle of the ring of 10 atoms along the [110] zone axis *y* = 0.1. The ^57^Fe Mössbauer spectroscopy for (c) *y* = 0 and (d) 0.1 at 5 K, respectively. Calculated interatomic magnetic exchange interactions on pyrochlore lattices (e) 16*d*-16*d*, (f) 8*a*-8*a* and 8*a*-16*d* for *y* = 0 and 0.1, respectively (interactions are given for three respective nearest neighbors (NN) shells).

To determine the hyperfine magnetic structure of Zr_0.75_Nb_0.25_Fe_2_Co_0.1_, the local Co occupation was first determined by atomic-resolution energy dispersive X-ray mapping using scanning transmission electron microscopy (STEM). It was observed that Zr and Nb atoms occupy the 8*a* site randomly. Interestingly, some weak intensity of Fe atomic columns was revealed on 8*a* sites as well (the white arrow in the top-right corner of Fig. 5a). For the Co atom, a preferred occupation is found on the pyrochlore-lattice 16*d* site, as shown in the bottom-left corner of Fig. 5a and Fig. S14. This suggests that the excess Co doping on the 16*d* site pushes the Fe atoms into the Zr/Nb-sublattice and antisite Fe emerges at the 8*a* site accordingly. Fig. 5b presents the integrated intensity profile mappings of the central Fe atomic column within the 10-atom ring structure. The obvious contrast observed between regions of higher intensity (red) and lower intensity (blue) within the atomic columns suggests a preferential clustering of Co atoms at the Fe 16*d* sites, leading to local chemical clustering within the pyrochlore lattice (see Figs S15 and S16 for details).

The ^57^Fe Mössbauer spectrum is obtained at 5 K to investigate the hyperfine magnetic exchange interactions of Fe-sublattice in Zr_0.75_Nb_0.25_Fe_2_ and Zr_0.75_Nb_0.25_Fe_2_Co_0.1_, respectively (see Fig. 5c and d and Fig. S17 for details). The fraction of the two magnetic sextets indicates an approximate ratio of 3 : 1 and reveals the interplanar ferromagnetic structure of Fe on pyrochlore-lattice along the (111) direction in Zr_0.75_Nb_0.25_Fe_2_ (Tables S5–S7) [44]. By comparison, there is an extra sextet in Zr_0.75_Nb_0.25_Fe_2_Co_0.1_ and the fraction is relatively small, which verifies the antisite Fe-8*a* due to Co doping. The other two sextets are for Fe in pyrochlore lattice and are similar to that of Zr_0.75_Nb_0.25_Fe_2_, i.e. interplanar ferromagnetic structure. By comparison, the content of one sextet decreases slightly from 66.7% to 63.7% and the magnitude agrees well with the Co occupation as determined by atomic-EDS, i.e. Zr : Nb : Fe : Co = 0.69 : 0.23 : 2 : 0.16 (Table S8).

To observe the multiple neighbor magnetic exchange interactions clearly, we also calculated the interatomic exchange interactions using the Green Function-based magnetic force theorem [24,45], as implemented in KKR-ASA formalism [46]. The additional Co atoms embedded in the 16*d* sublattice provide additional ferromagnetic interactions in the system. Both exchange interactions within both the Co and Fe pyrochlore-lattices are positive; the Fe_16_*_d_*-Fe_16_*_d_* is the strongest (Fig. 5e), which indicates that Zr_0.75_Nb_0.25_Fe_2_ and Zr_0.75_Nb_0.25_Fe_2_Co_0.1_ are typically ferromagnetic. The energy of the ferromagnetic configuration where the Fe on 8*a* sites is oriented parallel to the Fe and Co moments on 16*d* sites is much lower than the energy of antiparallel orientation (see Table S9 for details). The Co doping mostly affects the magnetic order due to weakening of the exchange interaction of pyrochlore-lattice and the introduction of extra magnetic exchange interaction (Fig. 5f). As the Co content continued to increase to 0.3, more ferromagnetic interactions were provided, resulting in a distinct increase in the saturation magnetization (Fig. S18).

### Correlation between the ZTE and magnetic order

**Figure 6.**
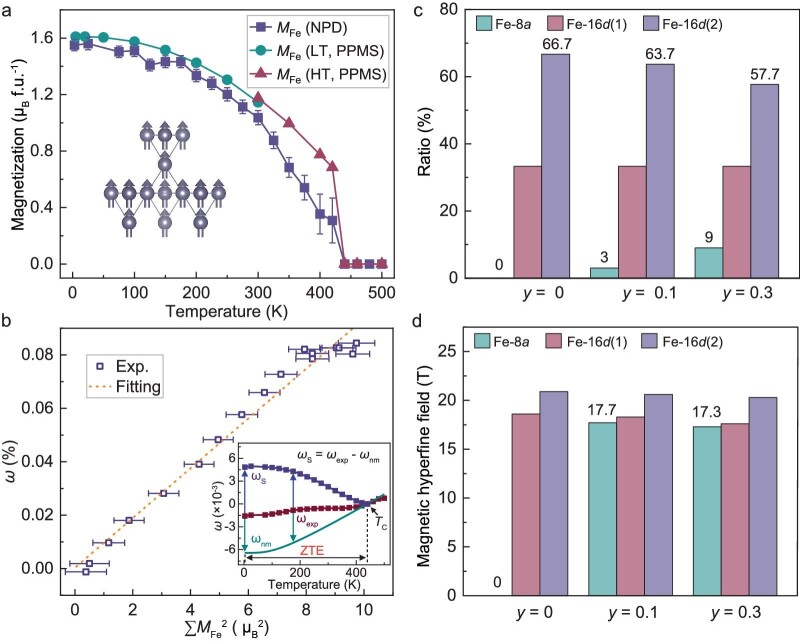
(a) Temperature dependence of Fe magnetic moments of Zr_0.75_Nb_0.25_Fe_2_Co*_y_* (*y* = 0.1) measured by NPD and Physical Property Measurement System (PPMS) (the inset shows the magnetic structure of cubic *y* = 0.1). (b) Positive correlation between the magnetic moment of the Fe lattice (|Σ***M***_Fe_|) and the spontaneous volumetric magnetostriction *ω*_S_ for *y* = 0.1 (the inset shows the calculation process for the *ω*_S_ based on experimental unit cell volume). (c) The ratios and (d) magnetic hyperfine field of Fe-8*a*, Fe-16*d*(1) and Fe-16*d*(2) in *y* = 0, 0.1, and 0.3, respectively, were observed at 5 K from the results of the ^57^Fe Mössbauer spectroscopy.

To reveal the evolution of magnetic ordering with temperature more clearly, the average magnetic moment of Fe/Co on pyrochlore-lattice, i.e. the 16*d* site, is determined by Rietveld refinements [47] on NPD patterns. The magnetic fraction on the 8*a* site is too weak to determine, and thus the magnetization of the matrix is dominated by the Fe/Co on pyrochlore-lattice. As shown in Fig. 6a, the magnetic moment at 3 K is ∼1.55 *μ*_B_/f.u., similar to the saturation magnetization of *M*-*H* curves at 5 K (1.61 *μ*_B_/f.u.), and decreases monotonously upon heating. In order to better see the extra magnetic contribution on thermal expansion, the nominal phonon effect on lattice is subsequently deduced by using the Debye–Grüneisen function (inset of Fig. 6b) [48]. The *ω*_exp_ is the experimental unit cell volume obtained from the temperature dependence of NPD. The *ω*_nm_ refers to the nominal phonon effect on lattice calculated by using high-temperature experimental lattice parameters [34,49]. Below *T*_C_, magnetic contribution emerges and increases gradually, yielding spontaneous bulk magnetostriction *ω*_S_ (*ω*_S_ = *ω*_exp_ − *ω*_nm_) [20,50]. Notably, the delicate balance between phonon and magnetic order leads to the apparent ZTE behavior [51,52]. The strong linear correlation between *ω*_S_ and ${{M}^2}( T )$ in Fig. 6b further identifies the *ω*_S_ increases with the stronger magnetic order of pyrochlore-lattice [8,53]. This correlation can be described by Landau theory as a function of *ω*_S_ and ${{M}^2}( T )$: ${{\omega }_S} = \kappa C{{M}^2}( T )$. Here, $\kappa $ is the compressibility constant, *C* is the magnetic volume coupling constant and ${{M}^2}( T )$ is the square of the value of the magnetic moment. In Fig. 6b, the ZTE is driven by the magnetic ordering of Fe and Co together. It can be seen that there is a positive linear correlation between *ω*_S_ and the square of ${{M}^2}( T )$. It can be added that the local composition heterogeneity induced by excess Co makes the separation into C1 and C2 magnetic. Thereby the magnetization as a function of magnetic field (*M*-*H*) curves changes more slowly below the magnetic ordering temperature, thus affecting ${{\omega }_S}$, as verified by macroscopic measurements of *M*-*H* and ZTE. As shown in Fig. 6c and d, the magnetic moments of Fe are stabilized by Co doping in the pyrochlore structure. Furthermore, the antisite Fe of 8*a* enhances the magnetic exchange interactions, leading to a higher *T*_C_ that maintains the *ω*_S_ and *ω*_nm_ balance over a wider temperature range, generating such ‘superior’ ZTE [54].

## CONCLUSION

We conclude on a quaternary pyrochlore metals Zr_0.75_Nb_0.25_Fe_2_Co_0.1_ with an isotropic ZTE of *α_a_* = +1.07 × 10^−6^ K^−1^ from 3 to 440 K. A comprehensive atomic-level crystal structure reveals a preferred occupation of Co atoms on pyrochlore-lattice substituted for Fe and the antisite Fe atoms on Zr/Nb-sublattice. High-resolution synchrotron X-ray diffraction shows that all the Bragg peaks split due to two cubic phases with different magnetic orders. *In-situ* neutron powder diffraction, Mössbauer spectroscopy and *ab initio* calculations indicate that the Co content in pyrochlore-lattice has weaker exchange interactions than that of Fe, yet the antisite Fe accounts for an extra positive exchange interaction between the 8*a* and 16*d* sites, thus enhancing the interplanar magnetic order yielded tuning thermal expansion. The present work highlights a large space on nonstoichiometric pyrochlore metals for developing the ZTE and related magnetic function materials.

## Supplementary Material

nwae462_Supplemental_File
