# Supplementary material for: Local chemical heterogeneity enabled superior zero thermal expansion in nonstoichiometric pyrochlore magnets
Source: Natl Sci Rev. 2024 Dec 17;12(3):nwae462. doi: 10.1093/nsr/nwae462 (PMC11835018; doi:10.1093/nsr/nwae462)
Supplement: nwae462_Supplemental_File [file nwae462_supplemental_file.pdf]

Supporting Information for

Local chemical heterogeneity enabled superior zero thermal expansion in nonstoichiometric pyrochlore magnets

Yanming Sun,[1,†] Ruohan Yu,[2,†] Sergii Khmelevskyi,[3] Kenichi Kato,[4] Yili Cao,[1,*] Shixin Hu,[5] Maxim Avdeev,[6,7] Chin-Wei Wang,[8] Chengyi Yu,[1] Qiang Li,[1] Kun Lin,[1] Xiaojun Kuang,[9,*] and Xianran Xing[1,*]

[1] Beijing Advanced Innovation Center for Materials Genome Engineering, Institute of Solid State Chemistry, Department of Physical Chemistry, University of Science and Technology Beijing, Beijing 100083

[2] The Sanya Science and Education Innovation Park of Wuhan University of Technology, Sanya 572000

[3] Vienna Scientific Cluster Research Center, Technical University of Vienna, Operngasse 10, Vienna A-1040

[4] RIKEN SPring-8 Center, Hyogo

[5] Institute of Applied Magnetics, Key Laboratory for Magnetism and Magnetic Materials of the Ministry of Education, Lanzhou University, Lanzhou 730000

[6] Australian Nuclear Science and Technology Organisation, Lucas Heights, New South Wales 2234

[7] School of Chemistry, The University of Sydney, Sydney, New South Wales 2006, Australia

[8] Neutron Group, National Synchrotron Radiation Research Center, Hsinchu 30076

[9] Guangxi Key Laboratory of Electrochemical and magnetochemical Functional Materials, College of Chemistry and Bioengineering, Guilin University of Technology, Guilin 541004

[†] These authors contribute equally.

*Corresponding authors (emails: yilicao@ustb.edu.cn kuangxj@glut.edu.cn xing@ustb.edu.cn)

EXPERIMENTAL SECTION

Synthesis. The $Zr_{1-x}Nb_xFe_2Co_y$ (x = 0.15, 0.25 and 0.35; y = 0, 0.1, 0.2 and 0.3) samples were prepared by arc melting in a high purity (≥ 99.999%) argon atmosphere using metal raw materials with purities higher than 99.9%. The samples were repeatedly melted more than 3 times in order to ensure a sufficient reaction and homogeneity of the composition. At the end of the arc melting process, the ingots are wrapped with molybdenum foil. They are then placed in a vacuum quartz tube for sealing and annealed at 1473 K for 1 week. Finally, to maintain the high temperature phase, the sample was quenched in water.

Characterization. Powder X-ray diffraction (XRD) were performed using an X-ray diffractometer (SmartLab 9 kw, Rigaku Corporation) with Cu Kα radiation. The linear thermal expansion curve ($\Delta L/L_0$) were measured on a thermal expansion meter (NETZSCH DIL402) in the temperature range 120-470 K. Define the coefficient of thermal expansion: $\alpha_l = \frac{(l-l_0)}{l_0 \times (\mathrm{T}-\mathrm{T}_0)}$. Here, ($\mathrm{T}$ - T_0) is the temperature window; l_0 and l are the initial and final sample lengths , respectively; α_l is the coefficient of linear expansion. Magnetization measurements were performed using a Physical Property Measurement System (PPMS, Quantum Design). A vibrating sample magnetometer (VSM) was used with a temperature rate of 8 K/min and a magnetic field rate of 100 Oe/sec. The ^{57}Fe Mössbauer absorption spectra were obtained in standard transmission geometry with ^{57}Co on rhodium basis. It was calibrated with α-Fe foil. The isomeric values were referenced to α-Fe. The 28 ml of 38 wt.% hydrochloric acid, 3.5 g of sodium chloride, and 4 g of sodium hydroxide were fixed to 100 ml with water to give final molar concentrations of 3.36 mol/L of hydrochloric acid solution, 0.6 mol/L of sodium chloride solution, and 1 mol/L of sodium hydroxide solution, respectively. In the Corrosion behaviour, $Zr_{0.75}Nb_{0.25}Fe_2Co_{0.1}$ is immersed in each of these three solutions. A GeminiSEM 300 (ZEISS, Germany) scanning electron microscope equipped with an Ultim Max (Oxford, Britain) EDS spectrometer was used for scanning electron microscopy (SEM) imaging and X-ray energy dispersive spectroscopy (EDS) elemental analysis. Atomic-resolution scanning transmission

electron microscopy (STEM) images, along with electron energy loss spectroscopy (EELS) and energy-dispersive X-ray spectroscopy (EDX) data, were acquired using a state-of-the-art FEI Themis TEM instrument equipped with a CEOS probe corrector and a Gatan image filter spectrometer. The microscope was operated at an accelerating voltage of 300 kV, with a probe convergence angle of 17.8 mrad and a probe current of approximately 45 pA for both STEM imaging and EELS/EDX acquisition. EELS analysis was conducted using a Gatan Quantum 965 GIF system, employing a dual EELS acquisition mode to simultaneously visualize the zero-loss peak and the Zr, Nb, Fe, Co edges for energy calibration purposes. The system achieved an energy resolution of approximately 0.9 eV, as determined by the full width at half maximum of the zero-loss peak. Temperature dependence of the synchrotron X-ray diffraction (SXRD) of sample were collected on the BL44B2 beamline (λ = 0.69995 Å) at SPring-8 (Japan). The temperature dependence of the neutron powder diffraction (NPD) was collected by the Australian Nuclear Science and Technology Organization (ANSTO) on ECHIDNA (λ = 2.44 Å). From the SXRD and NPD datas, Rietveld refinement using Fullprof software was used to obtain crystal and magnetic structure and lattice thermal expansion data.

SUPPLEMENTARY FIGURES

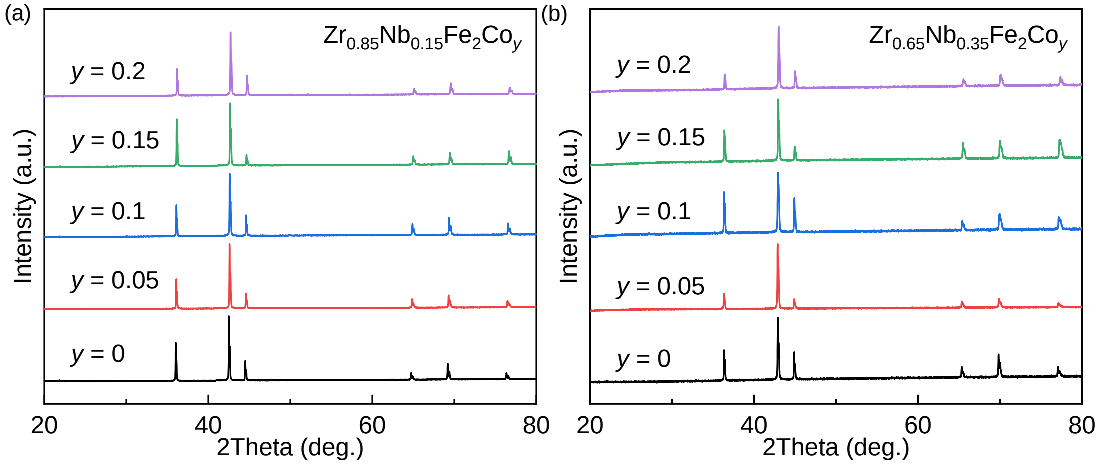

Figure S1. XRD patterns of $Zr_{0.85}Nb_{0.15}Fe_2Co_y$ and $Zr_{0.65}Nb_{0.35}Fe_2Co_y$ (y = 0, 0.05, 0.1, 0.15 and 0.2).

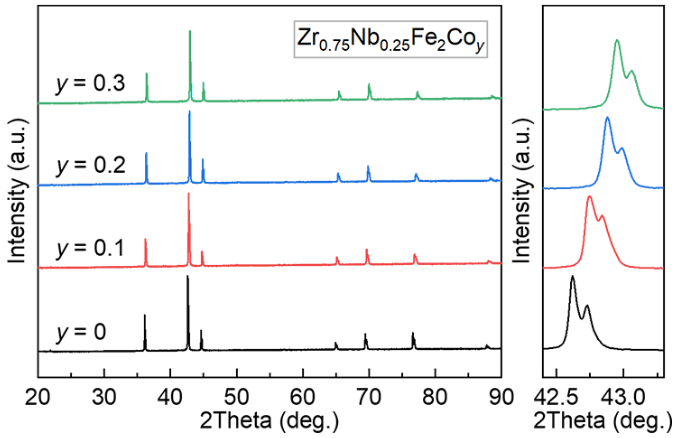

Figure S2. XRD patterns of $Zr_{0.75}Nb_{0.25}Fe_2Co_y$ (y = 0, 0.1, 0.2 and 0.3).

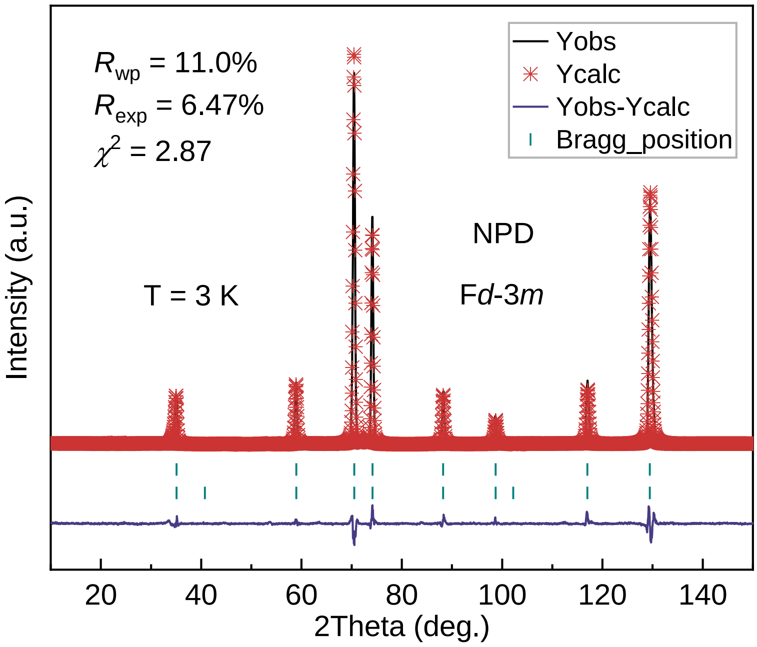

Figure S3 NPD refinement of cubic $Zr_{0.75}Nb_{0.25}Fe_2Co_{0.1}$ at 3 K.

(a) $Zr_{0.75}Nb_{0.25}Fe_2$

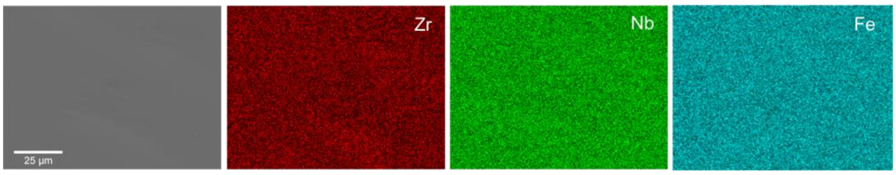

(b) $Zr_{0.75}Nb_{0.25}Fe_2Co_{0.1}$

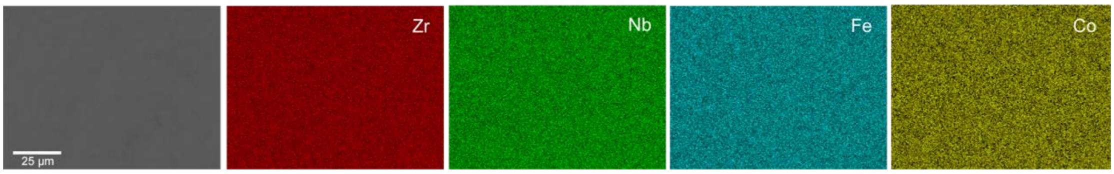

(c) $Zr_{0.75}Nb_{0.25}Fe_2Co_{0.3}$

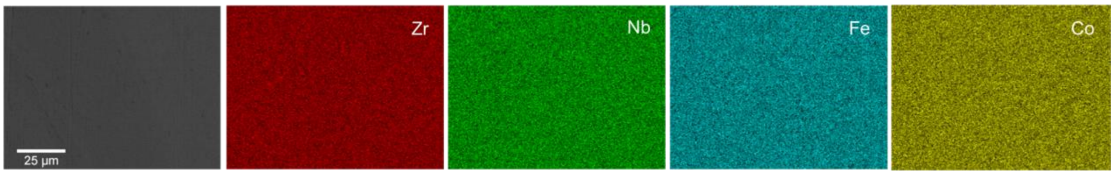

Figure S4. SEM images and EDS elemental maps of $Zr_{0.75}Nb_{0.25}Fe_2Co_y$ (y = 0 (a), 0.1 (b) and 0.3 (c)) samples of Zr, Nb, Fe or Co, which show uniform elemental distribution.

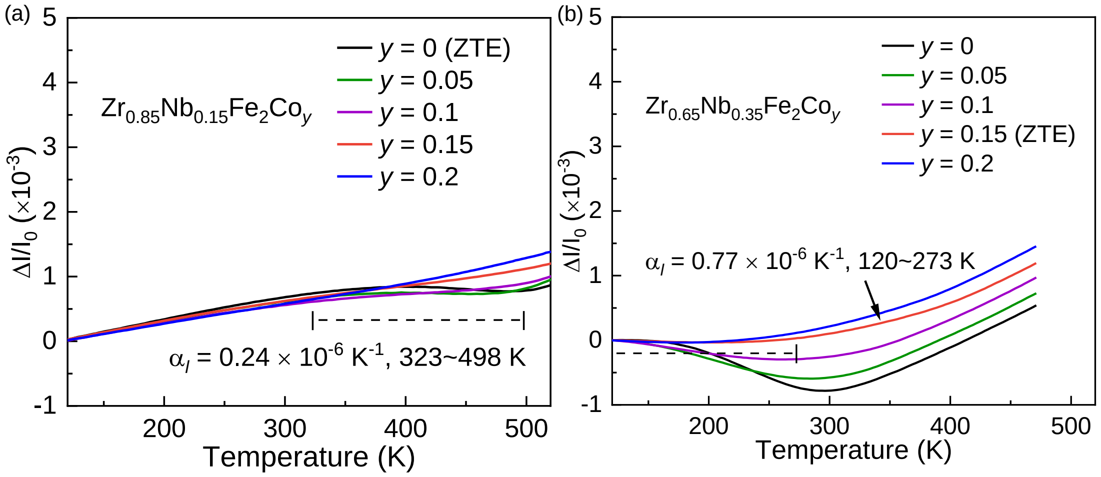

Figure S5. Linear thermal expansion ($\Delta I/I_0$) for $Zr_{0.85}Nb_{0.15}Fe_2Co_y$ and $Zr_{0.65}Nb_{0.35}Fe_2Co_y$ (y = 0, 0.05, 0.1, 0.15 and 0.2).

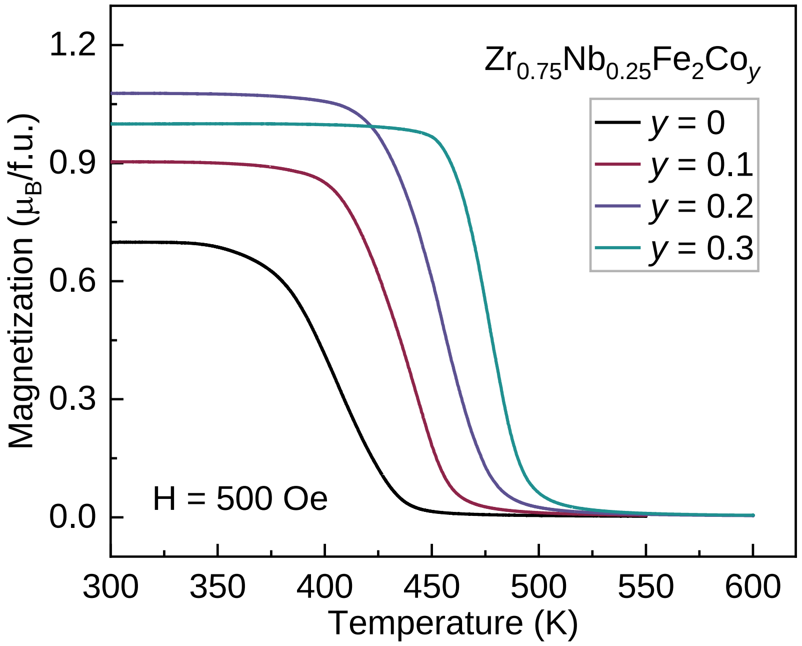

Figure S6. Temperature dependence of magnetization (*M*-*T*) for $Zr_{0.75}Nb_{0.25}Fe_2Co_y$ (y = 0, 0.1, 0.2 and 0.3) in zero-field cooling (ZFC) under an applied magnetic field of 500 Oe.

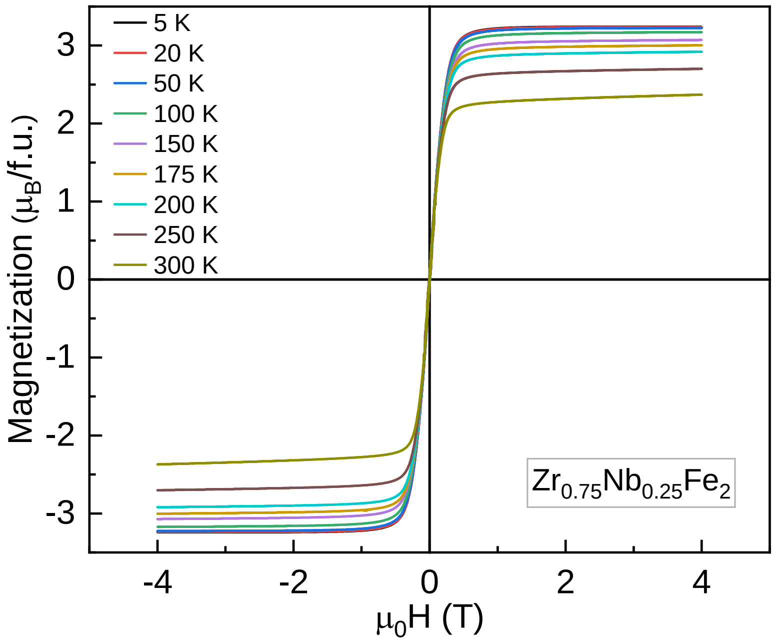

Figure S7. Isothermal *M-H* curves at 5−300 K for $Zr_{0.75}Nb_{0.25}Fe_2$.

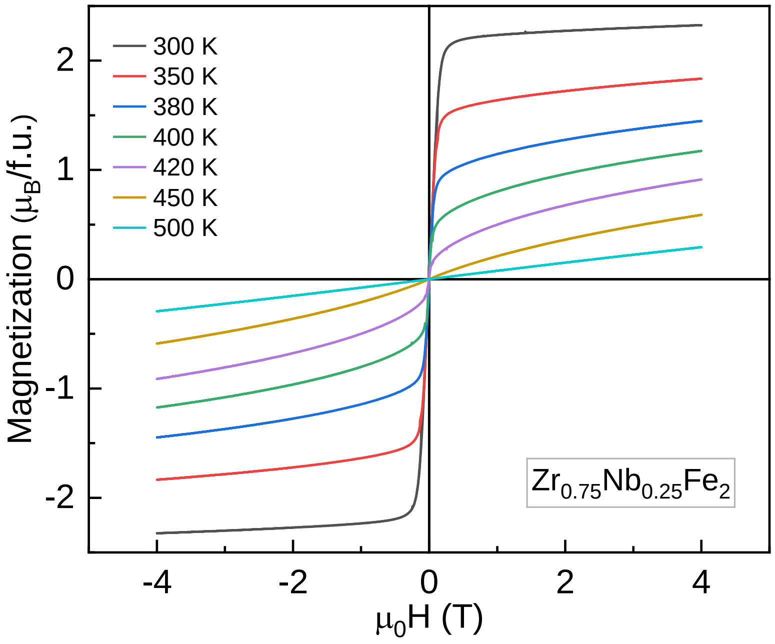

Figure S8. Isothermal *M-H* curves at 300−500 K for $Zr_{0.75}Nb_{0.25}Fe_2$.

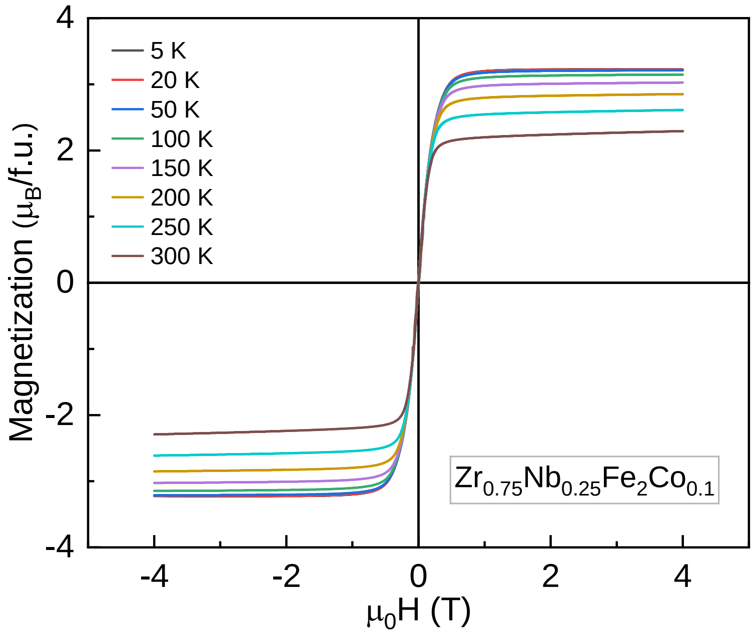

Figure S9. Isothermal *M-H* curves at 5−300 K for $Zr_{0.75}Nb_{0.25}Fe_2Co_{0.1}$.

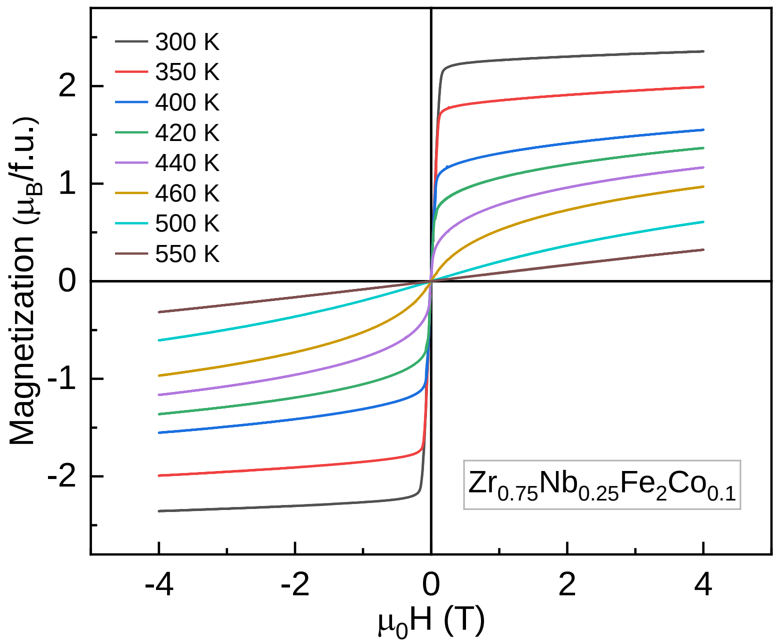

Figure S10. Isothermal *M-H* curves at 300−550 K for $Zr_{0.75}Nb_{0.25}Fe_2Co_{0.1}$.

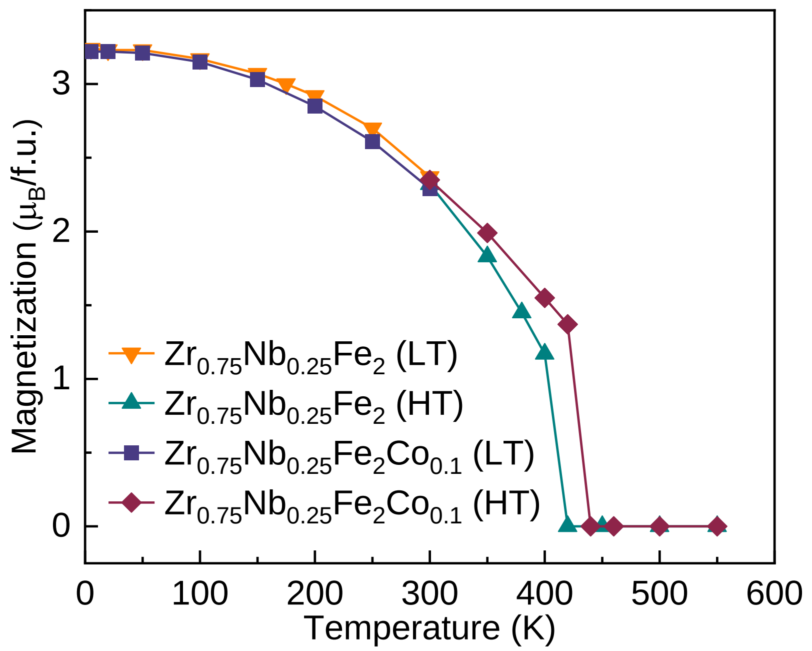

Figure S11. The saturation magnetization for $Zr_{0.75}Nb_{0.25}Fe_2Co_y$ (y = 0 and y = 0.1) under low and high temperature dependence by PPMS.

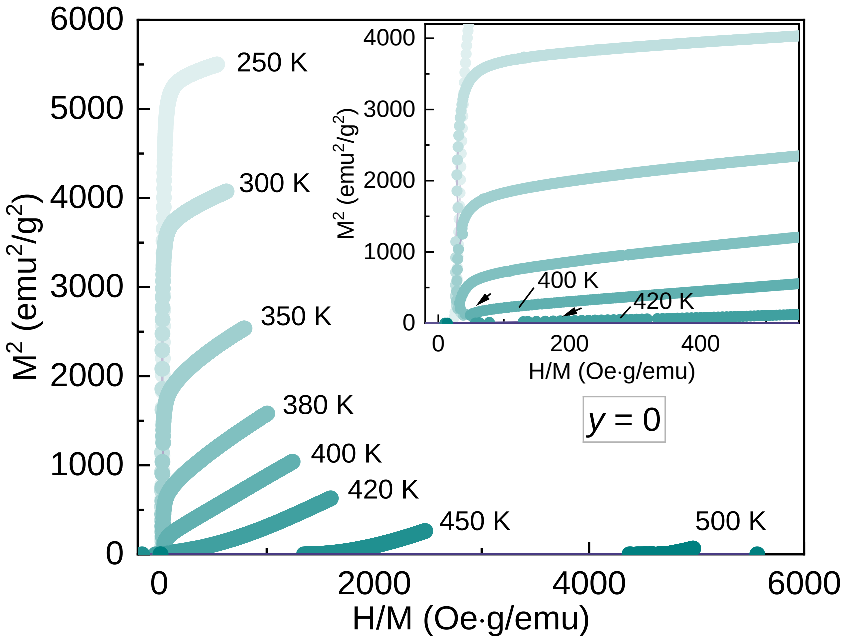

Figure S12. Isothermal Arrot plot of $Zr_{0.75}Nb_{0.25}Fe_2Co_y$ (y = 0) based on *M*-*H* curves.

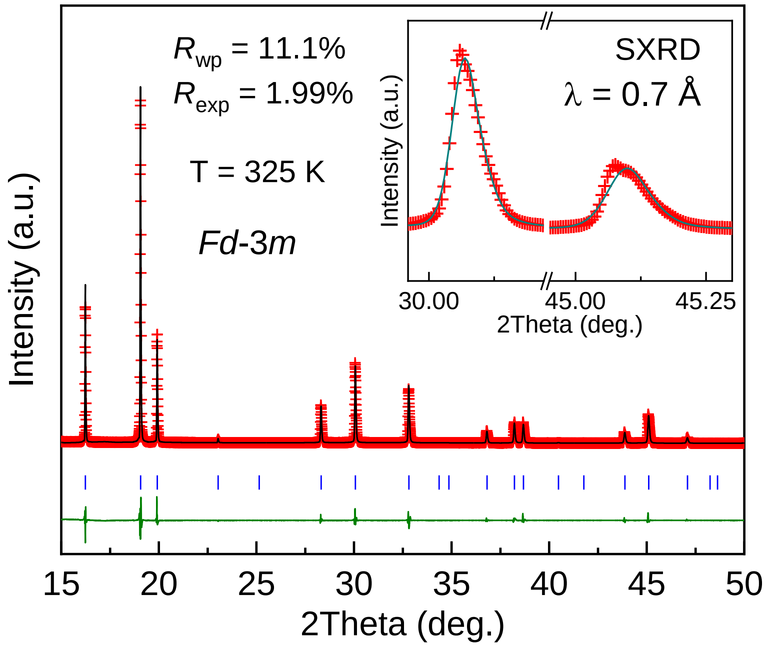

Figure S13. Single-phase rietveld refinement of SXRD patterns at 325 K for $Zr_{0.75}Nb_{0.25}Fe_2Co_{0.1}$.

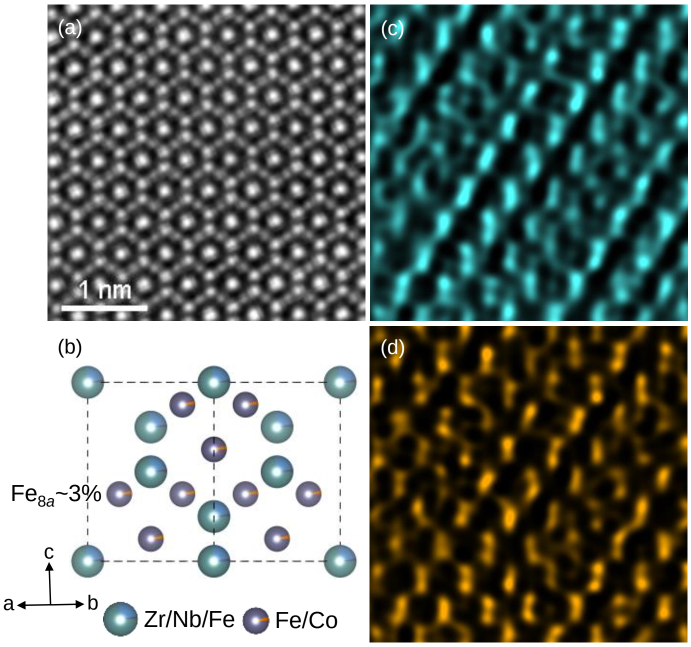

Figure S14. HAADF-STEM image along the [110] zone axis of $Zr_{0.75}Nb_{0.25}Fe_2Co_y$ (y = 0.1).

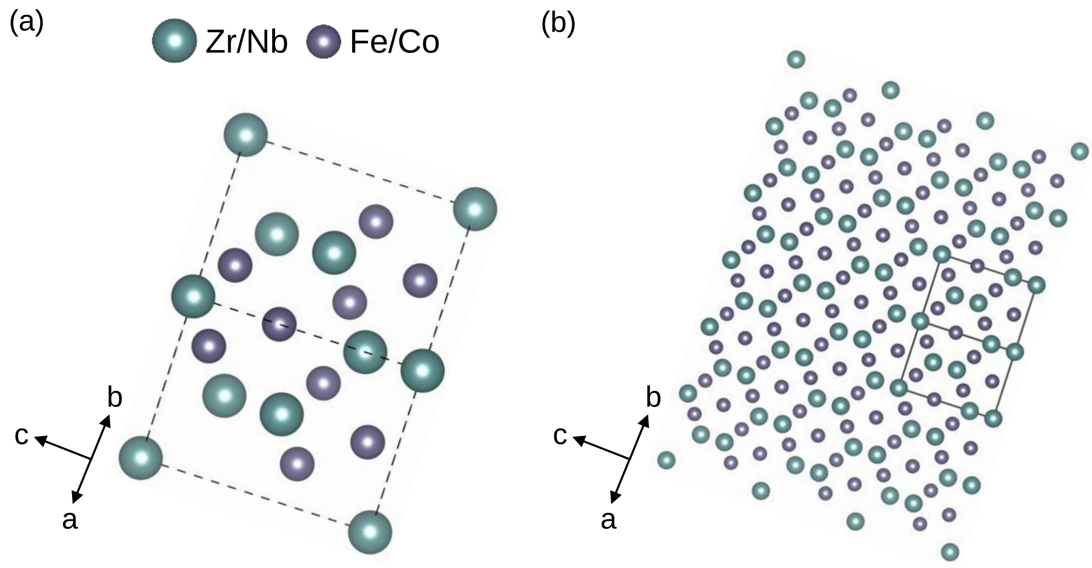

Figure S15. The cubic crystal structure of $(Zr,Nb)Fe_2Co_y$ in a pyrochlore lattice.

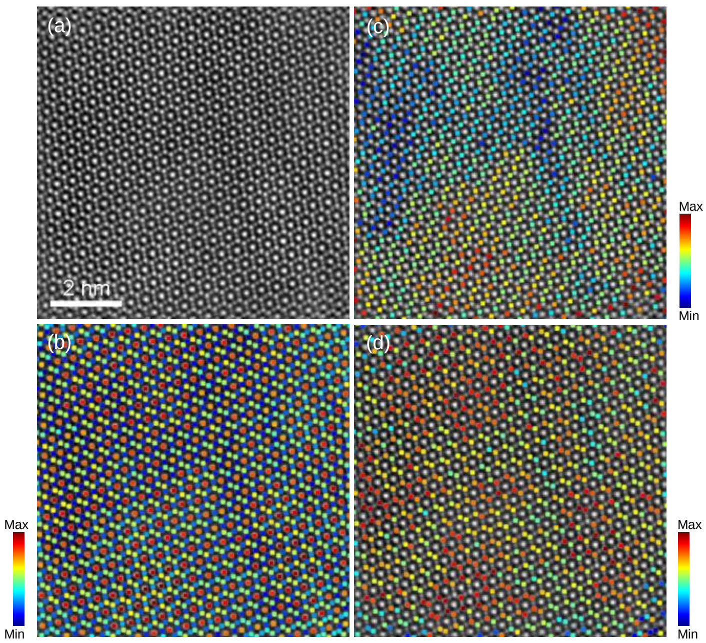

Figure S16. (a) HAADF-STEM image along the [110] zone axis of $Zr_{0.75}Nb_{0.25}Fe_2Co_y$ (y = 0.1); (b-d) The integrated intensity profile of the column of Zr/Nb or Fe/Co atoms.

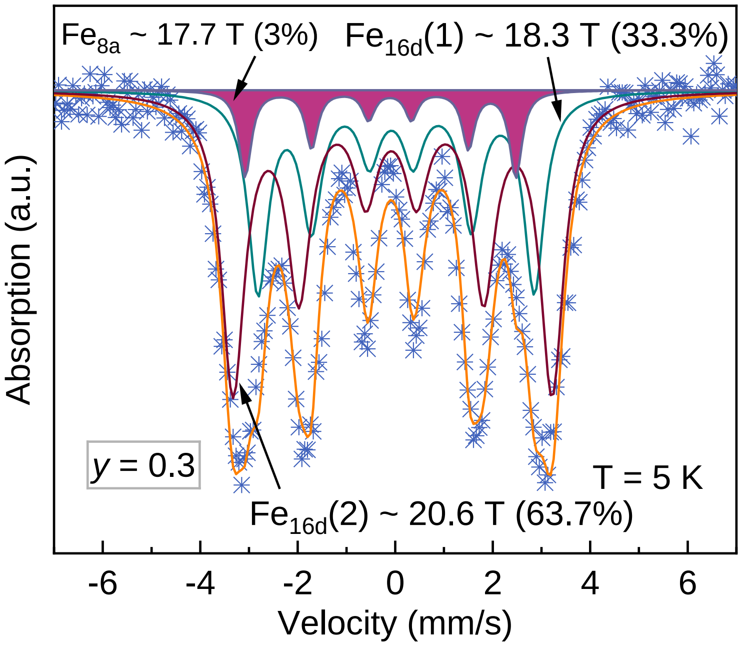

Figure S17. The [57]Fe Mössbauer spectrum of $Zr_{0.75}Nb_{0.25}Fe_2Co_{0.3}$ measured at 5 K.

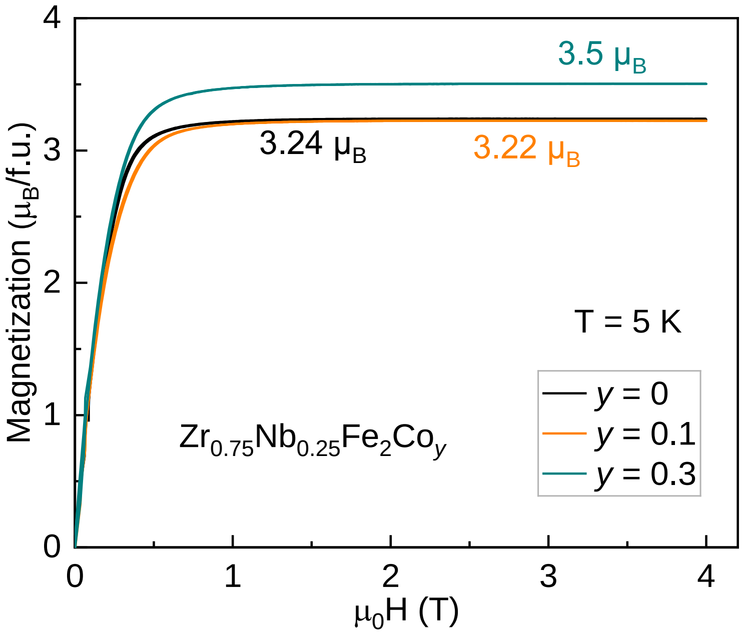

Figure S18. Isothermal *M-H* curves at 5 K for $Zr_{0.75}Nb_{0.25}Fe_2Co_y$ (y = 0, 0.1 and 0.3).

Table S1. Final refined structural parameters for cubic $Zr_{0.75}Nb_{0.25}Fe_2Co_y$ (y = 0.1) by neutron powder diffraction at 3 K.

Atom	Site	*x*, *y*, *z*	Occupancy	B_{iso} (Å^2)
Fe	16*d*	0.5, 0.5, 0.5	0.95	1.05(3)
Co	16*d*	0.5, 0.5, 0.5	0.05	0.61(3)
Zr	8*a*	0.125, 0.125, 0.125	0.73	0.61(3)
Nb	8*a*	0.125, 0.125, 0.125	0.24	0.61(3)
Fe	8*a*	0.125, 0.125, 0.125	0.03	0.61(3)

*a = 7.0135 Å and V = 344.984(1) Å^3, space group: *Fd*-3*m*, Z = 8; R_{wp} ~ 11%; χ^2 ~ 2.87.

Table S2. Temperature dependence of lattice parameters by NPD patterns for cubic $Zr_{0.75}Nb_{0.25}Fe_2Co_{0.1}$ compound (3–500 K).

Lattice parameter	a (Å)	unit cell volume (Å^3)	$\|M_{Fe}\|$ (μ_B/atom)
3	7.01347(1)	344.984(1)	1.55(4)
25	7.01375(1)	345.025(1)	1.56(4)
75	7.01382(1)	345.035(1)	1.50(4)
100	7.01408(1)	345.075(1)	1.51(4)
125	7.01446(1)	345.129(1)	1.41(4)
150	7.01483(1)	345.184(1)	1.43(4)
175	7.01524(1)	345.246(1)	1.43(4)
200	7.01547(1)	345.278(1)	1.33(4)
225	7.01562(1)	345.302(1)	1.28(4)
250	7.01573(1)	345.318(1)	1.20(4)
275	7.01579(1)	345.326(1)	1.11(4)
300	7.01586(1)	345.336(1)	1.04(5)
325	7.01584(1)	345.333(1)	0.88(5)
350	7.01586(1)	345.337(1)	0.68(6)
375	7.01603(1)	345.362(1)	0.54(8)
400	7.01644(3)	345.422(3)	0.35(14)
420	7.01693(3)	345.495(3)	0.31(15)
440	7.01721(1)	345.536(1)	0
460	7.01791(1)	345.640(1)	0
480	7.01874(1)	345.762(1)	0
500	7.01906(1)	345.809(1)	0

Table S3. Coefficient of thermal expansion CTE, temperature windows and temperature windows △T (K) for typical super-invar materials.

Super-invar materials	Coefficient of thermal expansion (CTE, $\times 10^{-6}$ K^{-1})	Temperature windows (K)	Temperature windows △ T (K)	Reference
$Zr_{0.75}Nb_{0.25}Fe_2Co_{0.1}$	1.07×10^{-6} K^{-1} (α_a)	3-440	437	This work
$Zr_{0.65}Nb_{0.35}Fe_{2.15}$	0.47×10^{-6} K^{-1} (α_a)	4-425	421	[1]
$Zr_{0.8}Ta_{0.2}Fe_{1.7}Co_{0.3}$	0.21×10^{-6} K^{-1} (α_l)	5-360	355	[2]
$Gd_{0.25}Dy_{0.75}Co_{1.93}Fe_{0.07}$	0.16×10^{-6} K^{-1} (α_l)	10-275	265	[3]
$LaFe_{13-x}Si_x$ hydrides	0.5×10^{-6} K^{-1} (α_l)	20-275	255	[4]
$LaFe_{10.3}Al_{2.7}$	0.4×10^{-6} K^{-1} (α_l)	4.2-250	245.8	[5]
$HoCo_2Mn_{0.5}$	-0.5×10^{-6} K^{-1} (α_l)	5-225	220	[6]
$Mn_3(Cu_{0.5}Ge_{0.5})N$	0.1×10^{-6} K^{-1} (α_l)	12-230	218	[7]
$Mn_3Zn_{0.93}N$	0.0583×10^{-6} K^{-1} (α_l)	10-185	175	[8]
$Zr_{0.8}Nb_{0.2}Fe_2$	0.3×10^{-6} K^{-1} (α_l)	298-470	172	[9]
$Fe_{63}Ni_{32}Co_5$	0.63×10^{-6} K^{-1} (α_l)	218-368	150	[10]
$LaFe_{10.4}Si_{2.4}$	0.8×10^{-6} K^{-1} (α_l)	15-135	120	[11]
$Mn_3Zn_{0.77}Mn_{0.19}N_{0.94}$	-0.52×10^{-6} K^{-1} (α_l)	5-110	105	[12]
$Mn_3(Cu_{0.55}Sn_{0.45})N$	0.5×10^{-6} K^{-1} (α_l)	255-323	68	[13]

Table S4. At 500 Oe, fitting results of the dM/dT of cubic kagome $Zr_{0.75}Nb_{0.25}Fe_2Co_{0.1}$ compound.

Peak number	Peak type	Wave area of fitted data	FWHM	Height max.	Peak weighted average center	Fit data peak area percentage
1	Gaussian	-4	19.05	-0.20	416.06	-20
2	Gaussian	-16	29.72	-0.51	440	-80

Table S5. At 5 K, fitting parameters of the ^{57}Fe Mössbauer spectrum of cubic kagome $Zr_{0.75}Nb_{0.25}Fe_2$ compound.

	Site	Isomer Shift (mm/s)	Quadrupole Splitting (mm/s)	Magnetic Hyperfine Field (T)	Ratio (%)
y = 0	Fe-16*d*(1)	-0.06	-0.08	18.6	33.3
	Fe-16*d*(2)	-0.07	0.08	20.9	66.7

Table S6. At 5 K, fitting parameters of the ^{57}Fe Mössbauer spectrum of cubic kagome $Zr_{0.75}Nb_{0.25}Fe_2Co_{0.1}$ compound.

	Site	Isomer Shift (mm/s)	Quadrupole Splitting (mm/s)	Magnetic Hyperfine Field (T)	Ratio (%)
	Fe-8*a*	-0.19	-0.4	17.7	3
y = 0.1	Fe-16*d*(1)	-0.04	0.05	18.3	33.3
	Fe-16*d*(2)	-0.05	0.04	20.6	63.7

Table S7. At 5 K, fitting parameters of the ^{57}Fe Mössbauer spectrum of cubic kagome $Zr_{0.75}Nb_{0.25}Fe_2Co_{0.3}$ compound.

	Site	Isomer Shift (mm/s)	Quadrupole Splitting (mm/s)	Magnetic Hyperfine Field (T)	Ratio (%)
	Fe-8*a*	-0.21	-0.2	17.3	9
y = 0.3	Fe-16*d*(1)	-0.03	0.1	17.6	33.3
	Fe-16*d*(2)	-0.07	0.03	20.3	57.7

Table S8. STEM-EDS elemental content of $Zr_{0.75}Nb_{0.25}Fe_2Co_{0.1}$.

Z	Element	Family	Atomic Fraction (%)	Atomic Error (%)	Mass Fraction (%)	Mass Error (%)	Fit error (%)	mole ratio
26	Fe	K	64.91	7.26	54.32	4.52	0.18	2.00
27	Co	K	5.12	0.83	4.52	0.65	0.19	0.16
40	Zr	K	22.53	3.55	30.8	4.27	0.49	0.69
41	Nb	K	7.44	1.17	10.36	1.44	1.26	0.23

Table S9. Calculated atomic moments, and total magnetization per f.u. in ferromagnetic state.

Magnetization intensity	$(Zr_{0.75}Nb_{0.25})Fe_2$	$(Zr_{0.75}Nb_{0.25})Fe_2Co_{0.1}$
$M_{Fe\text{-}16d}$	1.81	1.82
$M_{Co\text{-}16d}$	None	1.08
$M_{Fe\text{-}8a}$	None	2.89
$M_{Zr\text{-}8a}$	-0.50	-0.49
$M_{Nb\text{-}8a}$	-0.46	-0.46
M_{Total}/f.u.	3.13	3.18

We use Green Function Korringa-Kohn-Rostoker (KKR) band structure formalism and Local Spin Density Approximation[14]. The atomic disorder of Fe and Mn atoms on 12*j* and 4*f* sub-lattices was taken into the account using Coherent Potential Approximation (CPA) technique[15]. The energy of the ferromagnetic configuration where the Fe on Zr sites is oriented to parallel to the Fe and Co moments on 16*d* sites is much lower than energy of antiparallel orientation. However, the induced spin polarization polarization of the Zr and Nb atoms are opposite to the 3*d* metals moments in both cases $(Zr,Nb)Fe_2$ and $(Zr,Nb,Fe)(Fe,Co)_2$ alloys.

We calculate also the inter-atomic exchange interactions using Green Function based magnetic force theorem[16] as implemented in KKR-ASA formalism[17].

$$H = -\sum_{i,j\in 16d} J_{ij}^{Fe-Fe}\vec{e}_i^{Fe}\,\vec{e}_j^{Fe} - \sum_{i\in 16d;j\in 8a} J_{ij}^{Fe-Fe}\vec{e}_i^{Fe}\,\vec{e}_j^{Fe} - \sum_{i,j\in 16d} J_{ij}^{Fe-Co}\vec{e}_i^{Fe}\,\vec{e}_j^{Co} - \sum_{i,j\in 16d} J_{ij}^{Co-Co}\vec{e}_i^{Co}\,\vec{e}_j^{Co} - \sum_{i\in 16d;j\in 8a} J_{ij}^{Co-Fe}\vec{e}_i^{Co}\,\vec{e}_j^{Fe} - \sum_{i,j\in 8a} J_{ij}^{Fe-Fe}\vec{e}_i^{Fe}\,\vec{e}_j^{Fe}$$

Where $\vec{e}_i^{A}$ are the unit vectors of the spin moment directions on the corresponding lattice sites of *A*-atomic component.

The additional Co atoms embedded into the 16*d* subblatice provide and additional ferromagnetic interactions into the system which lead to the increase of the ferromagnetic Curie temperature (T_C), and also increase a total magnetization since both Co on 16*d* sublattice and anti-site Fe on 8*a* oriented parallel to the magnetization of the Fe on 16*d* positions. Lets note that the value of the antisite Fe atomic moments (see **Table S8** for details) is reaching a maximum values allowing by Fe 3*d*-metallic band splitting maintaining atomic charge neutrality in metallic alloys.

References

1. Sun Y, Cao Y and Hu S *et al.* Interplanar ferromagnetism enhanced ultrawide zero thermal expansion in kagome cubic intermetallic (Zr,Nb)Fe_2. *J Am Chem Soc* 2023; **145**: 17096-17102.
2. Li W, Lin K and Yan Y *et al.* A seawater-corrosion-resistant and isotropic zero thermal expansion (Zr,Ta)(Fe,Co)$_2$ alloy. *Adv Mater* 2022; **34**: 2109592. doi: 10.1002/adma.202109592
3. Hu J, Lin K and Cao Y *et al.* Adjustable magnetic phase transition inducing unusual zero thermal expansion in cubic RCo_2-based intermetallic compounds (R = rare earth). *Inorg Chem* 2019; **58**: 5401-5405. doi: 10.1021/acs.inorgchem.9b00480
4. Li S, Huang R and Zhao Y *et al.* Zero thermal expansion achieved by an electrolytic hydriding method in La(Fe,Si)$_{13}$ compounds. *Adv Funct Mater* 2017; **27**: 1604195.
5. Li W, Huang R and Wang W *et al.* Abnormal thermal expansion properties of cubic $NaZn_{13}$-type La(Fe,Al)$_{13}$ compounds. *Phys Chem Chem Phys* 2015; **17**: 5556-5560.
6. Fang CS, Wang JL and Hutchison WD *et al.* Controllable isotropic thermal expansion in series of designed magnetocaloric materials $HoCo_2Mn_x$ (x = 0-1.0). *J Alloy Compd* 2021; **863**: 158063.
7. Song X, Sun Z and Huang Q *et al.* Adjustable zero thermal expansion in antiperovskite manganese nitride. *Adv Mater* 2011; **23**: 4690-4694.
8. Wang C, Chu L and Yao Q *et al.* Tuning the range, magnitude, and sign of the thermal expansion in intermetallic $Mn_3(Zn,M)_xN$ (M = Ag,Ge). *Phys Rev B* 2012; **85**: 220103.
9. Shiga M and Nakamura Y. Magnetovolume effects and Invar characters of $(Zr_{1-x}Nb_x)Fe_2$. *J Phys Soc Jpn* 1979; **47**: 1446-1451.
10. Masumoto H, Kikuchi M and Sawaya S. Elastic anisotropy and its temperature dependence of single crystals of 63% Fe-32% Ni-5% Co alloy (super-Invar). *Transactions of the Japan Institute of Metals* 1970; **11**: 176-179.
11. Huang R, Liu Y and Fan W *et al.* Giant negative thermal expansion in $NaZn_{13}$-type La(Fe,Si,Co)$_{13}$ compounds. *J Am Chem Soc* 2013; **135**: 11469-11472.
12. Deng S, Sun Y and Wu H *et al.* Phase separation and zero thermal expansion in antiperovskite $Mn_3Zn_{0.77}Mn_{0.19}N_{0.94}$: An *in situ* neutron diffraction investigation. *Scripta Mater* 2018; **146**: 18-21.
13. Takenaka K and Takagi H. Zero thermal expansion in a pure-form antiperovskite manganese nitride. *Appl Phys Lett* 2009; **94**: 131904.
14. Perdew JP and Wang Y. Accurate and simple analytic representation of the electron-gas correlation energy. *Phys Rev B: Condens Matter* 1992; **45**: 13244-13249.
15. Ruban AV and Skriver HL. Calculated surface segregation in transition metal alloys. *Comp Mater Sci* 1999; **15**: 119-143.
16. Liechtenstein AI, Katsnelson MI and Antropov VP *et al.* Local spin density functional approach to the theory of exchange interactions in ferromagnetic metals and alloys. *J Magn Magn Mater* 1987; **67**: 65-74.
17. Ruban AV, Simak SI, Shallcross S *et al.* Local lattice relaxations in random metallic alloys: Effective tetrahedron model and supercell approach. *Phys Rev B* 2003; **67**: 214302.
